# Abiotic and biotic factors influence diapause induction in sexual and asexual strains of *Trichogramma brassicae* (Hym: Trichogrammatidae)

**DOI:** 10.1038/s41598-018-35626-7

**Published:** 2018-12-04

**Authors:** Somayeh Rahimi-Kaldeh, Ahmad Ashouri, Alireza Bandani, Nicolas Ris

**Affiliations:** 1 Department of Plant Protection, College of Agriculture and Natural Resources, University of Tehran, Karaj, Postcode: 31587-77871 Iran; 2 Institut Sophia Agrobiotech, INRA, CNRS, Université Côte d’Azur - 400, route des chappes – BP 167, 06903 Sophia Antipolis Cedex, France

## Abstract

The effects of some abiotic (maternal photoperiod and offspring developmental temperature) and biotic (host quality during both maternal and offspring generations) factors on diapause induction were investigated for two sympatric strains of *Trichogramma brassicae* Bezdenko (Hym: Trichogrammatidae) differing by infection status with regard to *Wolbachia*. The mode of reproduction, developmental temperature, maternal photoperiod and the quality of the host significantly affected diapause induction. The highest percentage of diapausing individuals were observed with the sexual strain reared at 14 °C using a “high quality” host and after a long photophase during the maternal generation. Environment-by-Environment, as well as Genotype-by-Environment interactions, was observed. All these patterns were discussed with the goal of providing relevant protocols for the commercial mass-rearing of such biocontrol agents. A successful diapause in beneficial insects could affect the efficiency of mass rearing by increasing the duration of storage conditions based on a high percent emergence and providing a large number of individuals at the appropriate time in the field season.

## Introduction

Egg parasitoids of the genus *Trichogramma* are widely used as effective biocontrol agents against many Lepidopteran pests, mainly because of the early destruction of their host before any plant damage, the ease of mass-rearing, and their short life cycle^1^. *Trichogramma* species can be divided into two groups according to their reproductive mode. The most common mode of reproduction is arrhenotoky, where males originate from unfertilized eggs and females from fertilized eggs. Thelytoky is another mode of reproduction in which unfertilized eggs produce females, and is either a property of the *Trichogramma* genome as in *T*. *cacoeciae* Marchal, or caused by *Wolbachia* infection^2,3^. Cytogenetic mechanisms of thelytoky in *T*. *cacoeciae* are typically apomictic which differs considerably from *Wolbachia*-induced parthenogenesis insect systems^4^. Thelytoky is of interest for mass-rearing and field efficacy, because it increases the proportion of females in a population. The presence of *Wolbachia* in *Trichogramma* species is not widespread with only 18 of 180 species tested known to be infected and while in some species *Wolbachia* infection is fixed at the population level (e.g. *T*. *oleae* and *T*. *cordubensis*)^5^, infection frequencies are low for some species (e.g. 6–26% of *T*. *kaykai* females^6,7^). As a consequence, both sexual-uninfected and asexual-infected individuals can be observed in a single species.

Because the *Wolbachia* infection not only alters the host reproduction but also may modify its overall physiology^8^, the advantage of thelytoky must be evaluated concomitantly with other biological features. Among them, the ability to diapause is of utmost importance for mass-rearing. Diapause is a period of arrested development during an insect’s life and other invertebrates during unfavorable environmental conditions^9^. Facultative diapause occurs during the prepupal stage in *Trichogramma* wasps and is known to be induced by various environmental factors including developmental temperatures^10–13^ and photoperiodic conditions during maternal pupal and adult development^14–16^. Because both *Wolbachia*’s metabolism and diapause may require significant and common physiological resources, they likely interact.

The present study was thus aimed at investigating the influence of maternal photoperiod, the direct influence of rearing temperature, and the influence of host quality on the diapause induction of two strains of *T*. *brassicae*, an uninfected-sexual strain and a *Wolbachia*-infected asexual strain (frequently reared and released for control of some key Lepidopteran pests in Iran)^17^. We have previously shown that both strains were able to overwinter in natural conditions (central Alborz Mountains) however sexual *T*. *brassicae* has more overwintering ability in natural climatic conditions^18^.

## Results

### Influence of the significant abiotic and biotic factors on the proportion of diapausing individuals

**Figure 1:**
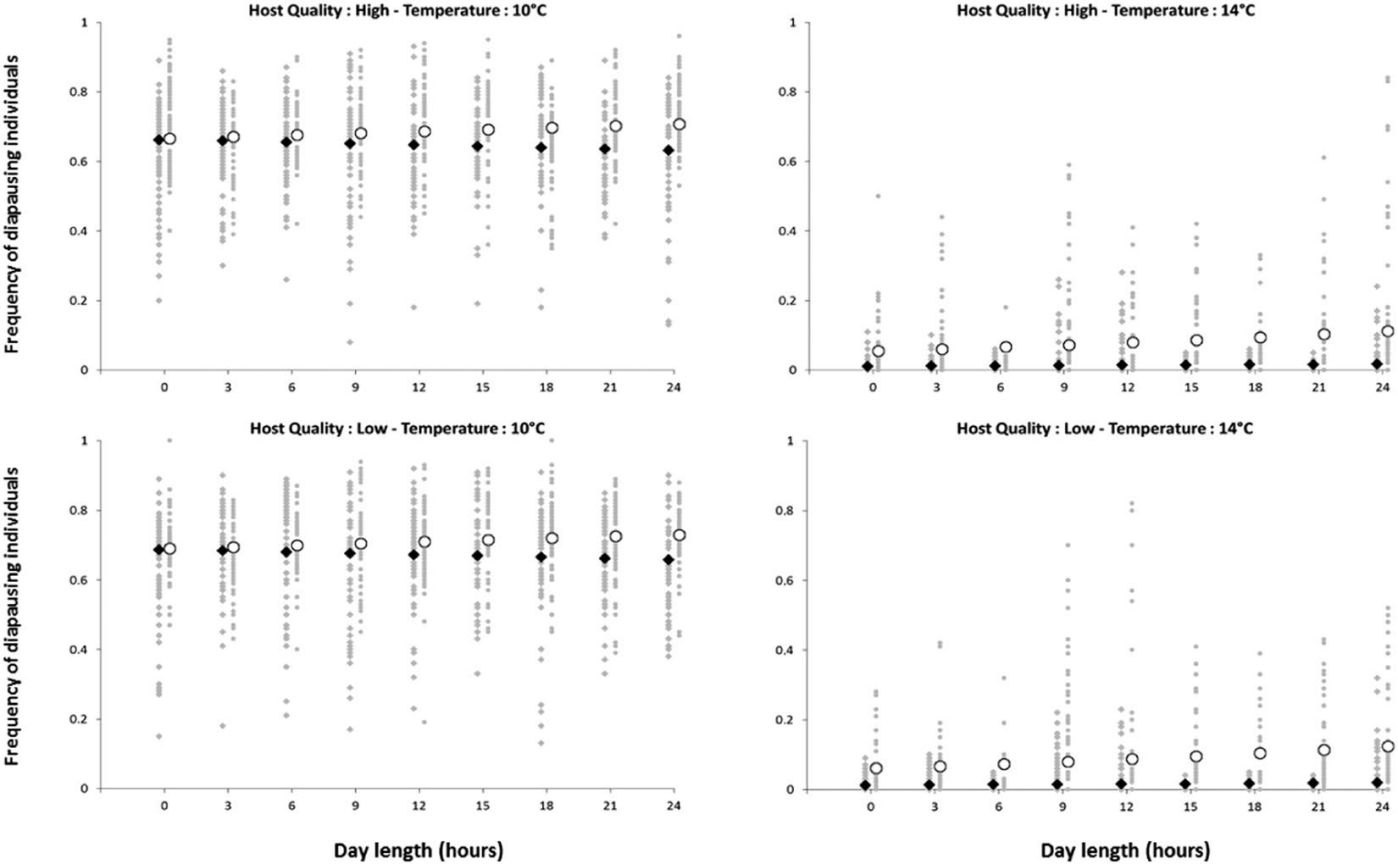
Influence of the significant abiotic (Temperature “10 and 14 °C” by Photoperiod “L: D = 0: 24, 3: 21, 6: 18, 9: 15, 12: 12, 15: 9, 18: 6, 21: 3 and 24: 0 hours” interaction) and (Offspring host quality “low and high” by *Trichogramma*’s strain “*Wolbachia*-infected and uninfected” interaction) factors on the frequency of diapausing individuals. For each graph, the predicted values of both the asexual (*Wolbachia* infected) and sexual (*Wolbachia* uninfected) *Trichogramma*’s strain are respectively indicated by black diamonds and white circles. All predicted values (grey diamonds) are surrounded by the observed values (circles).

As expected, the developmental temperature influences the induction of diapause with 67% and 5% of diapausing individuals at 10 °C and 14 °C, respectively. This temperature-dependent induction of diapause is however slightly modulated by the maternal photoperiod with almost no effect of this parameter at 10 °C but a positive correlation between the proportion of diapausing individuals and the duration of daylight at 14 °C (Fig. 1). The Offspring’s host quality also influences the proportion of diapausing larvae, this parameter being slightly increased with host of higher quality (Fig. 1).

The genotype-by-environment interactions are noteworthy, although both strains are prone to diapause at 10 °C (70% of diapausing regardless of other environmental conditions), differences were observed at 14 °C with 9% and 2% of individuals from the sexual and asexual strains entering diapause, respectively (Fig. 1). Moreover, the asexual strain did not seem to show a positive correlation to the photoperiod while the sexual one was (Fig. 1).

As shown in Table 1, the selected model included four main factors (“Strain”, “Temperature”, “Light” and “Offspring host quality”) and three interactions (“Strain × Temperature”, “Strain × Light” and “Temperature × Light”), almost all of them being highly significant. Although this situation would appear complex with genotype-by-environment interactions (Table 1, upper part) as well as environment-by-environment (Table 1, lower part), some general trends were seen (Fig. 1).

**Table 1:** Statistical analysis of the influence of experimental factors on the proportion of diapausing individuals.

	LR χ²	d.f.	*P*-value	
**Genotype-by-Environment**				
**Factor “Strain” and related interactions**				
Strain	0.3	1	0.61	
Strain x Temperature	1672.1	1	<0.001	***
Strain x Light	54.8	1	<0.001	***
**Environment-by-Environment**				
**Main factors and interactions only associated to environmental factors**				
Temperature	22407.0	1	<0.001	***
Maternal photoperiod	15.6	1	<0.001	***
Offspring Host Quality	66.2	1	<0.001	***
Temperature x Light	107.8	1	<0.001	***

***Is significant at 0.001 level.

## Discussion

More precisely, our results showed that the proportion of diapausing progeny was significantly dependent on developmental temperature. This result is thus consistent with previously published data obtained for other *Trichogramma* species^12–14,19–21^. The results point to a potential role of photoperiod in the diapause induction, depending on the temperature. Such a temperature-by-photoperiod interaction has been previously observed in *Trichogramma* wasps^12,14,19,21–23^. For instance, it has been demonstrated that most of the prepupae of *T*. *evanescens* Westwood went into diapause at 10 °C regardless of photoperiodic conditions, whereas the diapause induction at 15 °C was photoperiod-dependent^22^. In this case, the frequency of diapausing individuals was higher under short photophases (period of daylight) than under long ones. Previous results have also demonstrated that 100% of *T*. *principium* entered diapause at 12.5 °C regardless of the photoperiod experienced by the parental generation while the number of parasitoids that entered diapause increased with the decrease of the photoperiod at 15 °C^14^. More recently, others have shown that the photoperiod experienced by the maternal generation had an effect on diapause of *T*. *cacoeciae* at 13 °C but not at 10 °C^23^, which corresponds to our own observations on *T*. *brassicae*. The results also showed that the percentage of diapause was significantly higher at continuous light (L24:D0) both in sexual and asexual strains, suggesting that diapause increased in ultra-long photoperiod. These values at L24:D0 may not have ecological significance because the wasps never encounter this extreme condition, but have physiological significance which still remains to be explained^24^.

The third relevant abiotic factor is the quality of the host egg provided to the individuals which are of course of first importance in mass-production. Indeed, the storage of *E*. *kuehniella* (or other species) under cold conditions is a common way to secure the provisioning of hosts. The risks associated to the cold storage include (i) the decrease of water content, (ii) the degradation of some unstable components, (iii) the stiffening of the chorion. Increased host storage time for the Mediterranean flour moth, *E*. *kuehniella* caused a significant reduction in some biological characteristics of *T*. *brassicae*, such as fecundity, longevity, wing deformity and sex ratio^25^. The differences between the effect of host qualities on the diapause induction here were significant but the magnitude of the difference was small (37.2% and 35.7% of individuals with eggs of high and low qualities entering diapause, respectively). In contrast, it has been demonstrated that lower ability for determination of host quality in asexual *T*. *brassicae* could be the reason for the scarcity of *Wolbachia* infected *Trichogramma* in nature^26^, observed mortality was lower than expected in asexual *T*. *brassicae*. The test of prolonged durations of storage should be used to determine to what extent *E*. *kuehniella* eggs remain suitable not only for non-diapausing but also diapausing individuals.

The diapause percentage of asexual *T*. *brassicae* was found to be significantly lower than those of sexual strain (Fig. 1). These results suggest that *Wolbachia* infection disturbs the diapause of *T*. *brassicae* which consequently reduces the percentage of infected individuals in the nature. Although not in every case^27^, the *Wolbachia* infected thelytokous strain has been already observed in *T*. *oleae* Voegele and Pointel^28^ and in *T*. *brassicae*^18^ to be the less successful strain in comparison with sexual one. This pattern could be interpreted as the fitness cost for *Trichogramma* species harboring *Wolbachia*. Indeed, negative effects of the *Wolbachia* infection have been reported repeatedly for various traits including delayed egg hatching or adult emergence, parasitism and survival rates, adult size, discrimination ability (see for instance^26,29–33^). Although alternative or complementary explanations may occur (e.g. host-symbiont genomic conflicts^33^), such costs are mostly considered as the result of *Wolbachia* utilizing host resources for its own metabolism^8^. If so, the deprivation imposed by *Wolbachia* could drive the *Trichogramma* individual below a threshold under which the diapause induction is not possible. Of course, the slight decreases of diapause induction in the *Wolbachia*-infected asexual strain do not outweigh the concomitant benefit linked to the sole presence of “autonomous” females^34^. A previous study also found the mRNA levels of clock genes of *Wolbachia*-infected asexual *T*. *brassicae* strains were significantly lower than those of sexual strain, suggesting that *Wolbachia* infection suppresses clock gene expression in *T*. *brassicae*^35^. A more comprehensive investigation of pros and cons associated with the asexual strain would thus be relevant with regard not only to the performance in mass-rearing (growth rate, ability to diapause) but also the field efficiency.

Our results showed that the percentage of diapausing progeny was practically dependent to various abiotic and biotic factors. The diapause induction in *T*. *brassicae* could improve mass rearing methods by introducing the best strain, temperature, photoperiod and host quality to biocontrol agents’ manufacturers. As biocontrol agents have a relatively short life in comparison with pesticides, they must be produced shortly before they are used in augmentative releases. Thus diapause can be an efficient alternative method for increasing the life of these beneficial insects and to provide a sufficient supply of biocontrol agents at the appropriate time^36^.

## Methods

### Insects

The study was conducted with laboratory stocks (Ecology and Behavior Laboratory, University of Tehran) of two *Trichogramma brassicae* strains: one sexual and one asexual. Strains originated from parasitized eggs of European corn borer, *Ostrinia nubilalis* (Hubner) collected on maize in northern Iran (Baboulsar Region, south of the Caspian Sea; 36°42′N, 52°39′E) and the genetic background of two strains was previously evaluated by Kishani *et al*.^26^ to assume that any differences were as a result of the *Wolbachia* infection. Based on the size of nuclear ribosomal DNA (nrDNA) internal transcribed spacer 2 (ITS2) region, it has been concluded the strains were similar to each other and to other *T*. *brassicae* strains from the same northern region of Iran^26^.

Both strains were reared before the experiment for many generations (more than 100 generations) on *Ephestia kuehniella* Zeller eggs under constant laboratory conditions (25 ± 1 °C, L16: D8, 70 ± 5% r.h.). *E*. *kuehniella* eggs were obtained from a culture maintained at the Insectary and Quarantine Facility, University of Tehran (Tehran, Iran). Voucher specimens of both *T*. *brassicae* strains have been deposited at Zoology Museum (College of Agriculture and Natural Resource, University of Tehran).

### Detection of *Wolbachia* in *T. brassicae*

Before the experiment, the presence of *Wolbachia* in the asexual strain was checked by PCR with specific primers, “*Wolbachia* surface protein” (wsp)^37^. DNA from 50 frozen *T*. *brassicae* was extracted in a mixture of 30 µl of 5% Chelex solution and 2 µl of proteinase K (25 mg/ml), at 60 °C for 2 h followed by 15 min at 94 °C. Reaction cocktails for PCR amplification consisted of 2.5 µl of buffer 10 X PCR, 2.5 U of Taq polymerase, 2.5 mM MgCl2, 0.75 mM dNTPs, 1.5 µl of *wsp* forward primer (5′-TGGTCCAATAAGTGATGAAGAAAC-3′), 1.5 µl of *wsp* reverse primer (5′-AAAAATTAAACGCTACTCCA-3′) and 10 ng of DNA template in a final volume of 25 µl. The PCR conditions were: 94 °C for 3 min followed by 35 cycles at 94 °C for 1 min, 56 °C for 1 min, 72 °C for 2 min, and, finally, 72 °C for 10 min. The migration of the PCR product was realized using standard agarose gel and the expected size was consistent with that expected for *Wolbachia* (about 500 bp).

### Experimental design: principles

All the experiments were performed using eggs of *E*. *kuehniella* as the host.

**Figure 2:**
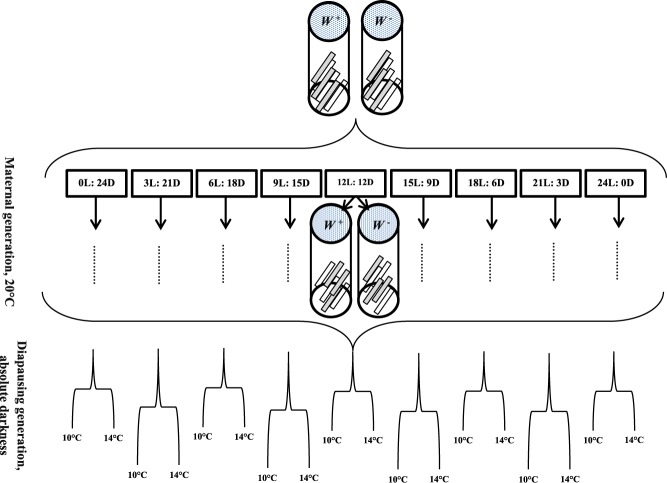
The experimental conditions in which maternal generation of both asexual and sexual *T*. *brassicae* reared on high-unshaded strips (less than 6 h old) and low-shaded strips (incubated at 4 °C for 120 h) host quality under different photoperiods of L: D = 0: 24, 3: 21, 6: 18, 9: 15, 12: 12, 15: 9, 18: 6, 21: 3 and 24: 0 hours at 20 ± 1 °C with r.h. 70 ± 5%. The diapausing generation of both asexual and sexual strains reared on high-unshaded strips (less than 6 h old) and low-shaded strips (incubated at 4 °C for 120 h) host quality under absolute darkness, 20 ± 1 °C and r.h. 70 ± 5%.

As shown in Fig. 2, we followed a fully-crossed design using five main factors that involved components of both the maternal and offspring environments:

1. the mode of strain reproduction (sexual vs. asexual).
2. the maternal photoperiod. The nine photoperiods (Light: Dark) tested were: 0:24, 3:21, 6:18, 9:15, 12:12, 15:9, 18:6, 21:3, 24:0 at 20 ± 1 °C and 70 ± 5% r.h.
3. the host quality in the maternal generation. Two *E*. *kuehniella* egg treatments (“High” and “Low”) were used. High quality *E*. *kuehniella* eggs were less than 6 h old and low quality *E*. *kuehniella* eggs were incubated at 4 °C for 120 h.
4. the developmental temperature of the offspring. Constant rearing temperatures of 10 °C or 14 °C, 70 ± 5% r.h. and absolute darkness was used.
5. the host quality in the offspring generation. The *E*. *kuehniella* egg treatments (“High” and “Low”) were used as previously described for the maternal environment.

Altogether, each of the two strains was successively exposed to 18 different maternal environments (9 photoperiods × 2 host qualities) followed by four different offspring environments (2 temperatures × 2 host qualities).

### Treatment exposure and rearing

For the first generation, two transparent plastic cylinders (height: 18 cm x diameter: 8 cm) were filled with either 500 females from the asexual strain or 500–1000 pairs (females and males) from the sexual strain; all individuals were about 24 h-old. A 20% honey water solution was sprayed on the internal surface of the container to feed the individuals. In each cylinder, “High” and “Low” quality *E*. *kuehniella* eggs were exposed to parasitism for 4 hours. To do so, 200–300 eggs were glued on cardboard paper strips (8 cm × 1 cm) using a non-toxic and water-soluble glue (Canco). A total of 36 strips, 18 of each host quality type, were placed in each cylinder. After exposure to parasitism, each egg card (with about 150–250 parasitized eggs) was separately placed in a clean tube and randomly assigned (within a host quality type) to one of the nine maternal photoperiod treatments. The maternal generation was held at a constant 20 °C and with a relative humidity (r.h.) of 70 ± 5%.

As soon as the mothers emerged (<24 hours old), they were placed in different transparent plastic cylinders (one for each combination of strain/ photoperiod/ maternal host quality). In each of the 36 cylinders, 120 cardboard paper strips, half with high and half with low quality *E*. *kuehniella* eggs (ca. 50 eggs per card) were offered to the females for parasitism for two hours (3–5 h after the light-on). Within each cylinder and for each offspring host quality treatment, the cardboard strips were split into two groups, one being kept at 10 °C, a favorable temperature for the induction of diapause in *Trichogramma* species^13,20^ and the other at 14 °C. Other environmental conditions were similar (70 ± 5% r.h. and absolute darkness). Each paper card was placed inside a glass tube (10 × 1.5 cm) separately, and each tube was closed with cotton.

At 14 °C, the darkening of parasitized host eggs occurred after 10 days and most of the individuals emerged after 45–50 days. Since *Trichogramma* females usually lay only one egg in each *E*. *kuehniella* egg^38^, the numbers of non-diapausing individuals were estimated by counting the numbers of eggs with an emergence hole. The remaining parasitized eggs were then dissected and living *T*. *brassicae* prepupae were counted and assumed to be diapausing individuals.

At 10 °C, the darkening of parasitized eggs occurs after about one month but no emergence was observed at three months after parasitism. We, therefore, considered these *T*. *brassicae* to be in diapause. All tubes were then transferred to 20 °C, L16: D8 and 70 ± 5% r.h. to break diapause and to facilitate the emergence of diapausing individuals. The number of diapausing individuals was estimated by counting the numbers of eggs with an emergence hole. The remaining parasitized eggs were then dissected to take into account the remaining living diapausing prepupae.

### Statistical analysis

The experimental design involved five environmental factors that were all crossed with one another: the “strain” (2 treatments: “sexual” and “asexual”), the “maternal host quality” (2 treatments: “High” and “Low” quality), the “offspring host quality” (2 treatments: “High” and “Low” quality), the “offspring developmental temperature” (2 treatments: 10 °C and 14 °C) and the “maternal developmental photoperiod” (9 treatments ranging from constant darkness to a constant light). The number of replicates per combination (n = 30) was initially equal.

Generalized Linear Models (GLM) were used to analyze the proportion of diapausing offspring (binomial distribution). The fit of the model was visually checked using residual plots obtained from the full model, all the main effects and interactions and rare outliers (4 in the final analysis were discarded). The most parsimonious model was then selected according to the Bayesian information criterion (BIC) criterion^39^ using a forward procedure and we finally realized an analysis of variance using the Likelihood-Ratio test (see Table 1). Residual plots for the selected model are given in the Supplementary Material.

Two sets of analysis were run, the “maternal developmental photoperiod” being treated firstly as a qualitative variable and then as quantitative one. The first analysis led to numerous significant interactions involving this variable but no real pattern (e.g. linear trend or threshold response). The results presented here are thus based on the second analysis (quantitative approach). All the analyses were performed using the software R, Rcmdr-package (version 3.0.3).
